# Investigating annotator bias in comment quality and incivility classification by formal education

**DOI:** 10.3389/frai.2026.1772844

**Published:** 2026-06-01

**Authors:** Lena Wilms, Anke Stoll, Marc Ziegele

**Affiliations:** 1 Faculty of Arts and Humanities, Heinrich Heine University Düsseldorf, Düsseldorf, Germany; 2 Faculty of Social and Behavioural Sciences, University of Amsterdam, Amsterdam, Netherlands

**Keywords:** annotator bias, content moderation, crowd annotation, deliberation, incivility, machine learning, online discussions

## Abstract

Machine learning models used in algorithmic content moderation in online discussions play an increasingly important role in detecting high-quality (e.g., constructive or engaging) and uncivil (e.g., toxic, hateful, or abusive) comments. Trained on human decisions, these models risk reproducing annotator bias, particularly when the perception of such nuanced categories is influenced by social backgrounds and training data lacks sufficient representational diversity. This study investigates the potential impact of annotators’ formal education on the classification of high-quality and uncivil user comments in online discussions. Using a dataset of 13,677 German user comments annotated by crowd workers with low, medium, and high formal education, we investigate divergences in classification outcomes when classifiers are trained on data annotated by different educational groups. In addition, we assessed statistical associations between fine-grained comment features and the annotations provided by each subgroup. Our results indicate that classifiers for high-quality and uncivil comments yield significantly different results when trained on data labeled by annotators with varying educational backgrounds. Furthermore, comment features used in traditional operationalizations of deliberative quality (e.g., solution proposals, additional knowledge) and incivility (e.g., vulgarity, accusation of lying) are more strongly associated with annotations by crowd annotators with medium and high formal education. We argue that such group-specific differences should be considered when developing machine learning models in both practice and research to reflect a more inclusive understanding of valuable and norm-violating user contributions to online discussions.

## Introduction

Online discussion platforms such as X, YouTube, and Instagram, as well as the comment sections of online news websites, increasingly rely on machine learning (ML) to support content moderation by automatically detecting comments that may require review or removal by human moderators (Gorwa et al., 2020). In recent years, significant efforts have been made to automatically identify so-called uncivil content, including hateful, discriminatory, or vulgar communication (Gehweiler and Lobachev, 2024; Hosseini et al., 2017; Risch et al., 2021; Stoll et al., 2020). At the same time, there is a growing interest in the classification of high-quality content under labels such as engaging (Risch and Krestel, 2020), constructive (Napoles et al., 2017), or deliberative (Behrendt et al., 2024; Falk and Lapesa, 2023), enabling moderators to encourage or highlight valuable comments to foster fruitful discussions (Stroud et al., 2015; Wang and Diakopoulos, 2021). However, the automated classification of such content poses severe challenges. One of the main obstacles is that the perceptions of those categories are to some extent subjective, as they are assessed according to people’s individual backgrounds, norms, and experiences (Herbst, 2010; Young, 2000). Such subjective judgments make classification susceptible to so-called *annotator bias*, which occurs when annotators’ perceptions differ systematically along different social backgrounds. For classifying high-quality and uncivil comments, this bias can manifest in annotators from varying social backgrounds systematically evaluating comments differently based on various communication features or language styles, such as the use of expressive communication, dialect or easy language (Sap et al., 2022; Young, 2000). This risk is high for crowd-annotated training data, which is created by freelance crowd workers, who are typically younger, predominantly male, and more highly educated than the general population (Posch et al., 2022).

For content moderation of online discussions, bias in comment quality and incivility classification can have serious consequences. Specifically, annotator bias can reduce the performance of classification algorithms for certain sub-populations if it fails to capture a holistic, socially inclusive picture of how comment quality and incivility are perceived. For example, by devaluing certain language styles, such bias may not only lead to a higher rate of false deletions of comments but also cause valuable contributions to be systematically overlooked by moderators when they seek content to encourage or highlight. Thus, bias may perpetuate discrimination and negatively affect the chances for minorities to express their views in online discussions. However, while there have been efforts to investigate annotator bias in the classification of uncivil comments and related concepts such as toxicity and offensive language (Al Kuwatly et al., 2020; Binns et al., 2017; Sap et al., 2022), to the best of our knowledge, no study has examined how annotators with different backgrounds perceive high-quality user contributions. Moreover, a systematic examination of annotator bias based on formal education is lacking, and its influence on the classification of high-quality and uncivil comments remains unclear.

Inspired by deliberation theory, we investigate possible differences along the level of formal education of crowd annotators, which has been discussed as a relevant factor in evaluating deliberative quality (Carstens and Friess, 2024; Sanders, 1997; Young, 2000), while also showing potential to advance the research on differing perceptions of incivility. Our study relies on a crowd-annotated dataset of 13,677 user comments from different German online discussions, containing balanced amounts of annotations by annotators with low, medium, and high formal education. In line with previous research in the field of annotator bias, we trained and evaluated several classification models on grouped, educationally distinct subsets (Al Kuwatly et al., 2020; Binns et al., 2017). To gain a better understanding of the rationale behind possible group-specific differences, we further calculate and compare statistical associations between the labeling of the three distinct groups with additionally annotated, fine-grained comment features of deliberation and incivility to investigate how relevant the occurrence of these features is for the collective judgment within the respective group.

With this study, we contribute to Natural Language Processing (NLP) and ML research on annotator bias by linking it to a rich body of social science research on deliberation and incivility. With the investigation of high-quality comments, we introduce a new subject to the research field of annotator bias. We also add to the research on annotator bias in comment classification by contributing insights into formal education as a potential bias risk and a relevant socio-demographic factor in the annotation of training data. We hope that the results of this study will be useful to researchers and practitioners working towards a more socially inclusive understanding of artificial intelligence (AI)-based systems for moderating online discussions.

## Theoretical underpinning: deliberation and incivility in political online discussions

### High-quality user comments

Alongside the detection of harmful content, there has been a growing interest in high-quality contributions to online discussions. Responding to such comments—either by highlighting them or actively engaging with them—has shown to increase the quality of subsequent discussions (Stroud et al., 2015; Wang and Diakopoulos, 2021), which makes it an attractive add-on in the toolkit of online moderation. To define high-quality comments, we use the concept of political deliberation as a theoretical underpinning, which is originally rooted in the theory of deliberative democracy. Within this framework, deliberative discourse forms an integral part of political will formation and is realized by a certain mode of communication that aims at a cooperative examination of political viewpoints, the free and equal negotiation of public will by arguments and reasoning, and the informed opinion formation by citizens (Habermas, 1984; Landwehr, 2012). Deliberative quality is an indicator to describe whether communication is suitable to facilitate this process: This is the case when communication is rational, reciprocal, and respectful (Bächtiger and Wyss, 2013). These deliberative quality criteria have also shown to align with the understanding of comment quality of online moderators (Diakopoulos, 2015; Loosen et al., 2017).

Recently, the concept of deliberative quality has also sparked interest among computer scientists (Behrendt et al., 2024; Falk and Lapesa, 2023). Related concepts from ML and NLP research to measure high-quality comments also align with deliberative principles like rationality, reciprocity, and respect. For example, Napoles et al. (2017) defined constructive comments as engaging, respectful, and/or informative conversations (ERICS), that are “intended to be useful or helpful” (Napoles et al., 2017, p. 15). Kolhatkar et al. (2020) “crowdsourced” their definition of constructive comments and found that discussion participants valued comments that were on topic, supported by evidence, and aimed at creating civil rather than emotional responses. Risch and Krestel (2020) defined comments that stimulate interactive discussion in news comment sections as engaging, and identify questions, additional information, personal stories, humor, and opinion expressed through agreement or disagreement as relevant comment features. In a later publication, Risch et al. (2021) used a broader definition to match dimensions of deliberative quality, such as rationality, reciprocity, and respect.

### Uncivil user comments

Automatically classifying uncivil comments is particularly important for online discussion platforms, as it supports compliance with legal obligations to remove illegal content and helps mitigate the negative impact of such comments on both readers and contributors (Chen et al., 2020; Gervais, 2015). Automated classification systems are employed to detect and remove uncivil content, assist moderators in responding more efficiently, and reduce the psychological burden associated with exposure to harmful language (Riedl et al., 2020). In the context of political online discussions, incivility is not merely characterized by a lack of communicative quality, but rather by the violation of social norms intended to disrupt or hinder constructive deliberation. The concept of political incivility can be understood as an umbrella term for various of such norm violations that undermine cooperative communication and that individuals perceive as worth sanctioning (Bormann et al., 2022). Often, this includes participants violating democratic norms of a free and equal political discourse (Papacharissi, 2004). It can further include norm violations of polite behavior or etiquette that challenge or damage discussion participants’ desired self-portrayal (Mutz and Reeves, 2005). Indicators for incivility in political online discussions include name-calling, pejorative utterances, and vulgarity as well as discrediting and excluding oppositional voices, discrimination, and threats to the physical integrity of others (Bormann et al., 2022). This operationalization has shown to match the perception of harmful and sanction worthy content of online moderators (Paasch-Colberg and Strippel, 2022; Stockinger et al., 2023) and shows significant overlap with concepts from NLP research, such as of toxicity (Hosseini et al., 2017; Risch et al., 2021), offensive language (Zampieri et al., 2019) and hate speech (Davidson et al., 2017).

### Formal education as a bias risk in comment classification

In the search of potential sources and subjects of annotator bias, deliberation theory provides valuable insights. Critical deliberation scholars put forward the idea that the assessment of deliberative quality is linked to people’s educational backgrounds (Sanders, 1997; Young, 2000). For example, Young (2000) claimed that the deliberative concept of a good discussion is rooted in an elitist idea of argumentation, which is often operationalized as mere ‘articulateness’—being able to present one’s standpoint in alignment with rules of formal discussion—and therefore linked to language conventions of formal education. Consequently, individuals with knowledge of and skills in formal discussion could not only engage in discussions more effortlessly but also find formally presented arguments more comprehensible. In Young’s opinion, this is especially true for highly educated, privileged segments of the population (Sanders, 1997; Young, 2012, 2000). Advocates of an inclusive deliberation concept even suggested that within certain societal groups a distinct understanding of deliberative quality could prevail. They argued in favor of acknowledging the value of communication modes from various political speech cultures, e.g., the expression of emotions, the sharing of personal experiences, and playful elements (Barkley Brown, 1994; Young, 2012, 2000). For instance, Polletta and Lee (2006) indicated that sharing personal stories is used by disadvantaged groups to voice their opinion and provide reasoning rather than using numerical and fact-based modes of argumentation traditionally connected to rational discourse.

In incivility research, an extensive body of literature dealed with the influence of personal characteristics and context on the perception of incivility (Bormann et al., 2022; Herbst, 2010). Empirical research indicated that not only the evaluation of various types of incivility but also their perceived severity varies based on attributes like age, sex, political orientation, and situational context (Kenski et al., 2020; Muddiman, 2017; Stryker et al., 2016). The assumptions from deliberation theory presented above also explain the potential impact of formal education on perceptions of uncivil communication. Following the line of argumentation on overvaluing certain language styles, Young (2000) stated that communication that does not align with elitist language runs the risk of being treated as less constructive or even uncivil. This may apply to easy language or misspellings, but also to colloquialism or dialect (Hayward, 2004; Sanders, 1997; Young, 2000). In their research on bias in toxicity classification, Sap et al. (2022) followed a similar reasoning, explaining differing perceptions of vulgarity through different tendencies towards a so-called ‘language purism’ attitude of the annotator. Additionally, critical studies from incivility theory pointed out that uncivil expressions may serve as a means for disadvantaged groups to articulate legitimate protest against prevailing social injustice—a point that is inspired by inclusive deliberation scholars like Young. Accordingly, for these groups, using uncivil communication to challenge society’s norms regarding acceptable behavior could be understood as a means of expressing discontent or even civil disobedience rather than norm violations (Edyvane, 2020; Masullo Chen et al., 2019), hinting to a group-specific understanding of incivility.

Such interpretations inform the assumption that people with different levels of formal education may judge both comment quality and incivility differently. With regards to the adoption of such concepts for ML purposes, researchers have explicitly emphasized that it may need further reflection on the inherent power dynamics within such definitions to reduce the risk of discrimination against minorities (Carstens and Friess, 2024; Miceli et al., 2022).

### Bias risks in crowd-annotated training data for algorithmic content moderation

Similar to deliberation research, NLP and ML works on annotator bias have recognized that social groups may systematically differ in how they perceive certain concepts, challenging conventional practices of standardized, aggregated measurements like *ground truthing* or *gold standards*, that are built on the assumption of a single, aggregated “true” value (Aroyo and Welty, 2015; Davani et al., 2022; Kathirgamalingam et al., 2024). For classification of incivility-related concepts, studies have shown that training classifiers on socially distinct subsets indeed results in systematic disparities in the outcomes of classification models. For example, Binns et al. (2017) created gender-specific training and testing datasets (meaning data which was labeled exclusively by either male or female annotators) from the Wikipedia detox project and compared the performances of those classifiers in a quasi-experimental approach. They found that all classifiers (male, female, and mixed) performed worse on exclusively female-labeled test data, leading to the assumption that women’s toxicity understanding is not reflected well. Using a similar approach, Al Kuwatly et al. (2020) expanded the analysis on various demographic attributes of the annotators. While the authors could not confirm any significant differences in hateful content classification by gender (specifically for personal attacks), they found systematic bias along other demographic categories, namely annotators’ first language, age, and education. Next to demographic factors, prior studies also identified bias along personal attitudes of the annotator, for instance along political orientation (Sap et al., 2022; Wich et al., 2020). Newer studies also indicate that bias could also occur along professional moderation expertise. For the use case of German online comment moderation specifically, Gehweiler and Lobachev (2024) showed that several ML approaches can effectively reproduce professional moderators’ decisions using datasets such as the One Million Post Corpus. However, Vecchi et al. (2025) underlined that the moderation behavior of untrained crowdworkers and professional moderators differ regarding both the selection of comments requiring moderation and in the use of different moderation techniques.

With regards to our research interest, two key aspects emerge from the state of research: First, although deliberation theory indicates socio-demographic differences in the perception of deliberative quality, there is a notable lack of studies addressing annotator bias in comment quality classification. To the best of our knowledge, there is no study to date that examines possible effects of socio-demographic backgrounds such as education on the classification of deliberative quality or other related constructs of high-quality comments. We address this research gap with the following research question: *How does formal education of crowd annotators influence the outcome of comment quality classification?*

Secondly, empirical results on educational level as a potential factor producing bias are largely lacking for both comment quality and incivility classification (or related concepts). As one of the few exceptions, Al Kuwatly et al. (2020) confirmed differing performances of ML classifiers for the use case of personal attacks using several educationally distinct training and test datasets. However, their study relied on the Wikipedia Detox corpus, which is skewed toward high educational levels and only allowed for a binary categorization of educational levels (above vs. below high school level). Such demographic imbalance is a known challenge in crowd-working environments, as it is in line with observations by Posch et al. (2022) that crowd workers in Germany are more highly educated than the general population. Since these study results might be limited by the underrepresentation of people with low levels of formal education within their participant population, we argue that a more granular inspection is necessary. We address this research gap with the following research question: *How does formal education of crowd annotators influence the outcome of comment incivility classification?*

## Data and method

To address our research questions, we compiled a crowd-annotated dataset of online comments labeled for the classification of comment quality and incivility. Unlike previous studies, our dataset provides balanced amounts of annotations from crowd workers of low, medium, and high formal education for all comments, enabling a quasi-experimental research design. To explore possible differences between classifiers trained on educationally distinct annotator groups, we followed a two-step analysis strategy (Al Kuwatly et al., 2020; Binns et al., 2017): First, we examined whether classifiers trained on the annotations of distinct educational groups differed systematically in their classification results. Second, we analyzed the statistical associations between comment features of deliberative quality and incivility with the group-specific, overall labels, using a subset with fine-grained manual coding.

### Compilation of the crowd annotation dataset

#### Sample

This study drew on an extensive database of German online comments from two news media comment sections (social media and online forum) and two online citizen participation projects. The comments stem from political online discussions between November 2017 and November 2021. They contain conversations around either political events covered by news media or municipal legislation in Germany. For the final sample, we randomly selected up to 4,000 comments from every discussion provider. When datasets contained less than 4,000 comments, all comments were selected. After removing duplicates and moderation comments, our final sample contained 13,677 comments, including 7,872 comments from news media comment sections and 5,805 comments from online citizen participation projects.

#### Annotation task description

The data annotation was conducted in collaboration with the German crowdsourcing provider *Clickworker* from January 2023 to July 2023. Three groups of crowd annotators of different educational levels completed annotation tasks on the identical dataset of 13,677 comments. During the annotation process, crowd annotators labeled comments along the categories *High-Quality* (yes/no) and *Uncivil* (yes/no). Our annotation instructions were informed by various operationalizations of high-quality and uncivil comments to ensure theoretical grounding in deliberative quality and incivility while allowing for individual interpretative flexibility that is necessary to capture subjective interpretations of annotators. We also decided against using the terms ‘deliberative’ or ‘uncivil’ within the instructions, since these terms may not be familiar to most people in Germany. Accordingly, a comment is considered as high-quality if annotators evaluated it as *completely or partially enriching*. This included *comments that add arguments, proposals, or new perspectives to the discussion, or in some other way help users find it stimulating or valuable*. A comment is labeled as uncivil if annotators evaluated it as *completely or partially inappropriate*. This included *comments that are rude, abusive, hateful, or in any other way make users feel disrespected*.^1^ Note that the categories are not mutually exclusive. This resulted in a database of 123,091 annotations (with nine annotations of different crowd workers for 13,677 comments^2^).

#### Determining crowd annotators’ level of education

To address the limitations of existing datasets used for educational bias inspection, the study created a novel dataset characterized by a balanced distribution of annotations by crowd workers of different formal educational levels. We collected nine annotations per comment for each comment quality and incivility. Three annotations each were made by crowd workers of the same educational level, which was identified as either low, medium, or high. To do this, we set up three identical tasks containing all 13,677 comments, with one task each being exclusively reserved for crowd workers with either low, medium, or high educational level. To participate in the task, crowd workers undertook a pre-screening to determine whether they met the entry requirements (matching educational level and passing a German language test on the crowd annotation website). To determine the level of formal education, we used the highest formal degree in general and academic education of the crowd annotators and ranked them according to the Standard Classification of Education 2011 (UNESCO, 2012). Crowd annotators assigned to the educational level ‘low’ completed secondary general or intermediate schools up to grade 10 (ISCED 0–2). Crowd annotators assigned to the educational level ‘medium’ completed intermediate schools up to grade 13 that led to higher education entry qualification (ISCED 3–4). Finally, crowd annotators assigned to the educational level ‘high’ held an academic degree (university degree or equivalent, ISCED 5–8). By ensuring an equal distribution of annotations across these educational levels, our study provides a more robust database for inspecting the influence of formal education on comment quality and comment incivility classification.

#### Annotator information and group comparison

A total of 681 crowd annotators participated in the annotation for this study. When comparing annotators by level of formal education, the largest group consisted of those with a high level of formal education (*n* = 256), who provided an average of 160 annotations per person. This was followed by annotators with a medium level of education (*n* = 228), who provided an average of 178 annotations per person. The smallest, but most active, group consisted of annotators with a low level of education (*n* = 197), who averaged 207 annotations per person. We also asked crowd annotators to provide information on additional socio-demographic information, including age, gender identity, and the frequency with which they read and write online comments, to assess task familiarity. Table A1 in the appendix provides further information on the sample.

Following a quasi-experimental research logic, we assumed that differences in the annotation of high-quality and uncivil comments and the resulting classification results can be attributed to differing educational levels, if other annotator characteristics and task familiarity did not significantly differ between the educationally distinct groups. To test whether our collected data meets these requirements, we performed F-tests on participants’ age, as well as chi-square tests for the variables gender identity and the frequency of writing and reading online comments. Results indicated that there were no significant differences between members of the educational groups regarding their gender identity or the frequency of writing and reading online comments. However, the F-test and subsequent *post hoc* comparisons (Scheffé) revealed that the group of medium-educated annotators was significantly younger than both the low- and high-educated groups, *F*(2) = 15.50, *p* < 0.001. *Post-hoc* tests showed a mean age difference of −4.5 years between the low and medium groups (*p* < 0.001), and −6.1 years between the medium and high groups (*p* < 0.001). We take this difference into account in both our analytical approach and the limitations section.

#### Label aggregation and annotator agreement

For each comment, nine annotators of three educational levels assigned a total of nine labels. For the analysis, we aggregated the three labels of each group (low, medium, and high formal education) to one common label using majority vote (median). This way, we created three congruent datasets for comment quality and incivility, divided by the educational level of the annotators. Finally, we aggregated an intergroup ‘mixed’ value for comparison. Here, we included one annotation from each group, which were selected randomly (Al Kuwatly et al., 2020; Binns et al., 2017). To measure intercoder reliability, we calculated group-specific Krippendorff’s alpha values for each educational group as well as the annotations in the mixed value (Krippendorff, 2004).

Table 1 shows that intercoder agreement for comment quality is low within the three groups (Krippendorff’s alpha values between 0.29 and 0.39; percentage agreement between 64.47 and 69.40 percent), implying heterogeneity regarding the annotation of comment quality. Within the group of low formal education, annotator agreement is the lowest (Krippendorff’s alpha = 0.29, percentage agreement = 64.47). Among all three groups, label distribution is almost balanced, with the group of medium formal education annotating the most comments a high-quality (51 percent), followed by high (47 percent) and low formal education (46 percent). Table 2 shows that for incivility, intercoder-agreement is low within the three groups as well (Krippendorff’s alpha values between 0.23 und 0.33; percentage agreement between 77.65 and 83.27 percent). Further, it is noticeable that only a small number of comments have been annotated as uncivil overall (between 11 and 13 percent among the three groups), resulting in a strongly imbalanced distribution of incivility in the dataset. This imbalance is likely to have a major impact on classification performance, which we will address in the results and limitations sections.

**Table 1:** Label distributions and agreement per educational level for comment quality.

Metric	Low education	Medium education	High education	Mixed
*N*_HighQuality_	6,277 (46%)	6,885 (51%)	6,499 (47%)	6,560 (48%)^a^
*N*_NotHighQuality_	7,400 (54%)	6,792 (49%)	7,178 (53%)	7,117 (52%)^a^
Krippendorff’s alpha	0.29	0.39	0.39	0.35^a^
Percentage agreement	64.47%	69.40%	69.38%	67.39%^a^

a Value is calculated based on three annotations per comment using randomly selected annotations from one group each.

**Table 2:** Label distributions and agreement per educational level for comment incivility.

Metric	Low education	Medium education	High education	Mixed
*N*_Uncivil_	1,729 (13%)	1,515 (11%)	1,584 (12%)	1,542 (11%)^a^
*N*_NotUncivil_	11,948 (87%)	12,162 (89%)	12,093 (88%)	12,135 (89%)^a^
Krippendorff’s alpha	0.23	0.33	0.31	0.27^a^
Percentage agreement	77.65%	83.27%	81.89%	80.37%^a^

a Value is calculated based on three annotations per comment using randomly selected annotations from one group each.

### Analysis design and classification

#### Quasi-experimental classifier comparison

To answer the research questions of how formal education of crowd annotators may affect the outcome of comment quality and incivility classifiers, we trained and tested classification models based on the distinct educational groups, employing a quasi-experimental 4 × 4 train-test design (three educational groups and one mixed). This way, we could investigate to what extent the classifiers trained on the different groups were able to match annotations across the groups. We assumed an educational annotator bias if the classification results (performances) of the different models differed systematically between the test sets.

As classification models, we employed the publicly available German BERT model *bert-base-german-dbmdz-uncased via* Hugging Face^3^ as a state-of-the-art baseline commonly used in comment classification tasks (Devlin et al., 2019). The model is pretrained on a large and heterogeneous German-language corpus, including Wikipedia, news data, and web text (over 2 billion tokens in total), which supports robust language representations for downstream classification tasks. We used the uncased variant to reduce sparsity in token representations and improve generalization across informal user-generated text. For each annotator group, the model was fine-tuned separately using the respective training subset of crowd-annotated data. Input texts were tokenized using the model’s original tokenizer (lowercasing enabled), with a maximum sequence length of 512 tokens and a batch size of 32, following the recommendations of the original paper (Devlin et al., 2019). Fine-tuning was performed using the AdamW optimizer with a learning rate of 2e-5 for 3 epochs. Fine-tuning was conducted on a Quadro RTX 8000 GPU without modifying the underlying model architecture or tokenizer, and no additional pretraining or data augmentation was applied. To obtain robust performance estimates, we applied 20-fold cross-validation, resulting in 20 independent training and evaluation runs per annotator group and task. In each fold, the model was trained on 95% of the data and evaluated on the remaining 5%, ensuring strict separation between training and test data. Model performance was assessed by comparing predicted labels with the crowd-annotated majority votes for both comment quality and incivility. We report accuracy and macro-F1 scores *averaged* across all folds. To assess whether performance distributions differ significantly between groups, we applied the Kolmogorov–Smirnov test to the fold-wise macro-F1 scores (*N* = 20 per group). To ensure reproducibility, the code used for all analyses is publicly available on GitHub.^4^

#### Statistical associations of comment features on a manually coded subset

In a second step, we investigated whether different comment features traditionally used for the operationalization of deliberation and incivility were influential for labeling across different educational groups (Behrendt et al., 2024; Binns et al., 2017). We used a convenience subsample of 1,540 comments from our dataset that had been labeled for the occurrence of these fine-grained features within a quantitative content analysis by three trained student assistants in a previous research project (Heinbach and Wilms, 2022). The dataset includes annotations of categories tied to deliberative quality: Arguments, additional knowledge, solution proposals, polite salutation, expression of mutual respect, and storytelling. It further contains information on the occurrence of uncivil comment features: contempt, screaming, vulgar language, insults, accusation of lying, sarcasm.^5^ To identify possible patterns within annotators’ perceptions of high-quality and uncivil comments, we calculated logistic regressions between the occurrence of the communication feature in a comment (0 = *no*; 1 = *yes*) and the majority votes of the educationally distinct annotator groups regarding perceived comment quality and incivility (0 = *no*, 1 = *yes*). As additional variables, we included comment length as well as age (both 0/1 coded for better comparison^6^) to the analysis. We added the latter to control for the different distribution of age within the educationally distinct groups.

## Results

### Classifier comparison

#### Comment quality classification

**Figure 1:**
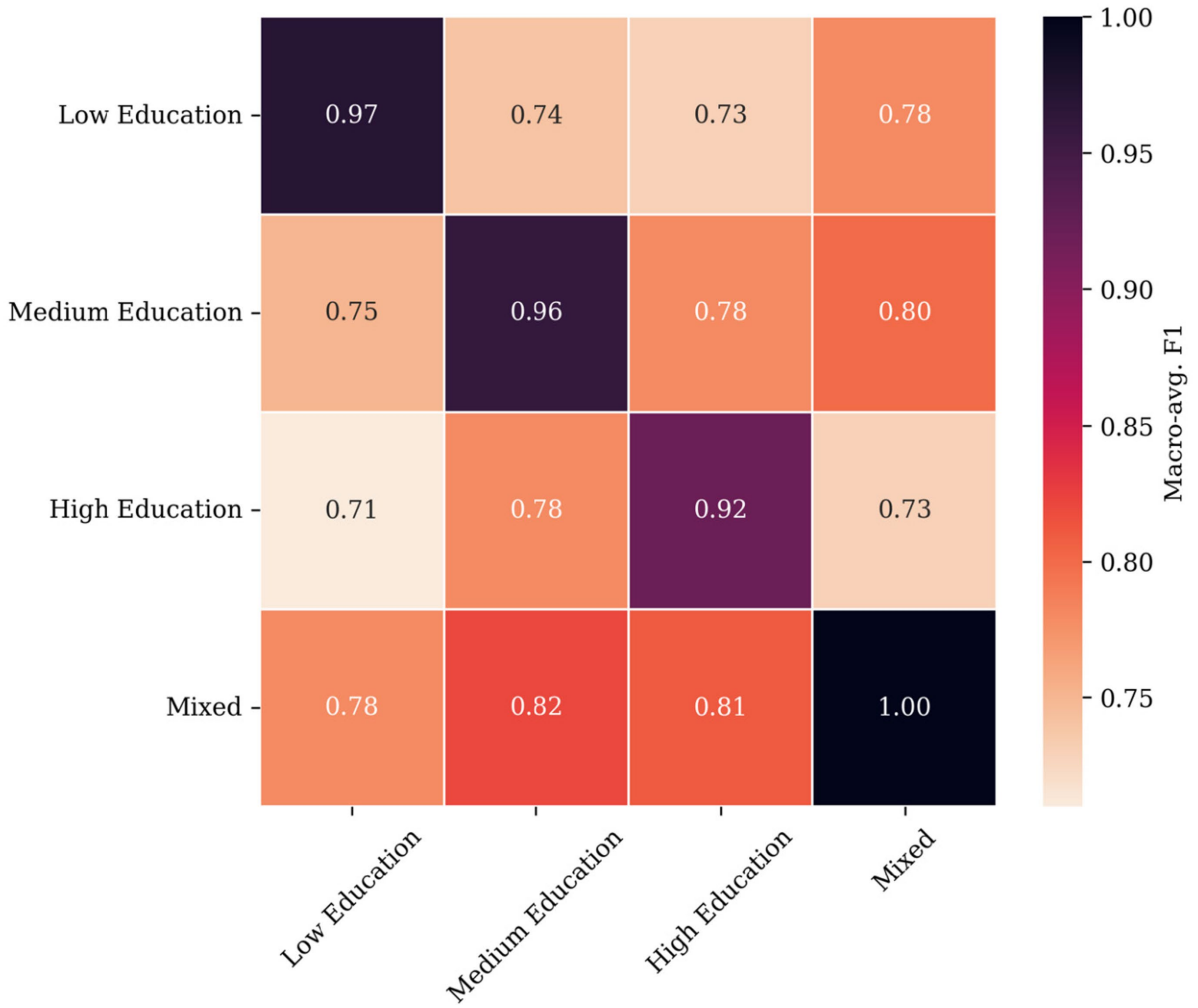
Macro-average F1-scores for comment quality classification by educational group. Performance scores are averaged values of 20 runs (20-fold cross validation). *N*_train_ = 12,993 and *N*_test_ = 684 comments in each run.

The results indicate that classifiers trained on annotations from distinct annotator groups yield significantly different performance outcomes. Figure 1 gives an overview of the model performances (macro-average F1-scores) of the four classifiers on the four different test datasets. Table A3 in the appendix presents a breakdown of model performance metrics.

The classification results show clearly that classifiers trained on distinct educational groups performed best when applied on a test set that stems from the same group. For train-test pairings within a group, overall model performance is very high with macro-average F1-score and accuracy of over 0.9, indicating that most of the comments were classified correctly and that the classifiers were able to learn a robust pattern based on the data labeled within each group. Despite low intercoder agreement within each group (Krippendorff’s alpha ranging from 0.29 to 0.39), the heterogeneity of annotations did not appear to adversely affect overall classification performance when aggregated. However, in this study design, very high performances can be partly attributed to the use of 20-fold cross-validation, increasing the likelihood of overfitting and inflating performance metrics (see discussion section). Nonetheless, classifiers applied on test sets of the other groups performed significantly lower. Kolmogorov–Smirnov tests confirmed significance of performance deviations.^7^ To summarize, classifiers for comment quality trained on data labeled by educationally distinct annotator groups achieved significantly different results. Further, within-group classifications achieved remarkably high and clearly better performances than classifications on test data from the other groups.

#### Comment incivility classification

**Figure 2:**
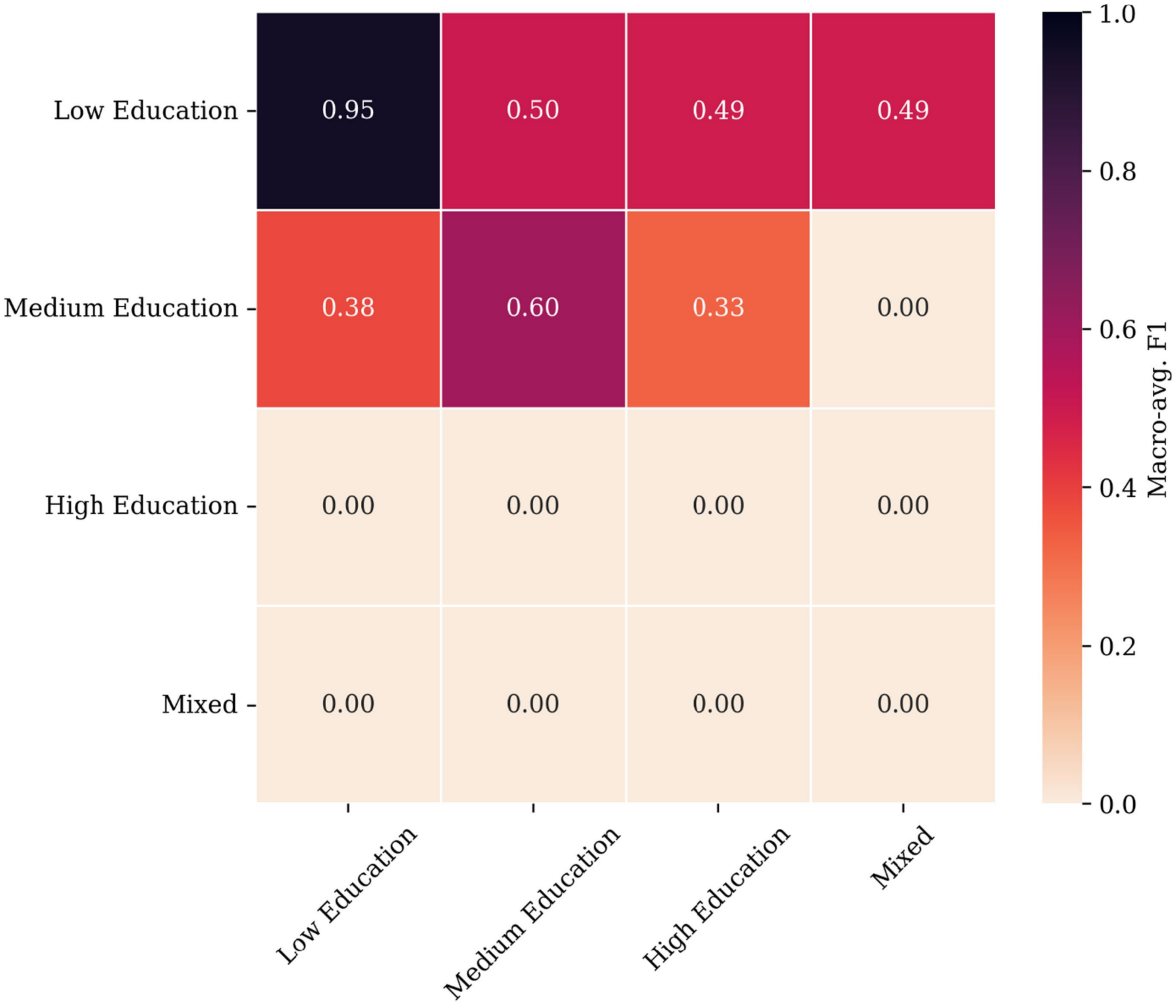
Macro-average F1-scores for incivility classification by educational group. Performance scores are averaged values of 20 runs (20-fold cross validation). *N*_train_ = 12,993 and *N*_test_ = 684 comments in each run.

Similarly, for incivility, the results indicated that classifiers trained on annotations from the distinct groups yield different outcomes. Figure 2 gives an overview of the overall model performances (macro-average F1-scores, see Table A4 in the appendix for a breakdown of model performance metrics).

While the results indicated a similar pattern to comment quality classification, they were strongly affected by overall class imbalance. For the low education group, classifiers trained and tested on annotations from this group performed significantly better (macro-average F1-score = 0.95) than when applied to the data from the medium (macro-average F1-score = 0.5) and high education groups (macro-average F1-score = 0.49) as well as mixed (macro-average F1-score = 0.49).^8^ For medium education, this pattern was similar but not as pronounced here (macro-average F1-score between 0.0 and 0.6). However, notably, classifiers trained on annotations from the high-education as well as from the mixed group achieved average F1-scores of 0.0 across the 20 runs on all test sets. A similar tendency was also observed, though less consistently, for the medium-education group. This pattern likely reflects the interaction of severe class imbalance and small subgroup-specific fold sizes: when uncivil comments are very sparse, some train–test splits may contain too few informative positive examples for the classifier to learn a stable decision boundary, leading it to default to the majority class. We therefore interpret the all-zero scores not as a presentation artifact, but as evidence that the subgroup-specific incivility task became too sparse and unstable for reliable classification under the present cross-validation setup. In sum, the results of the incivility classification point to a possible group-specific classification pattern. However, the imbalance between uncivil and non-uncivil comments in the dataset significantly impacted model performances and interpretability, as in many cases, the models failed to predict incivility altogether.

### Statistical associations of comment features

In a second analysis step, we investigated how the occurrence of fine-grained deliberative and uncivil features was related to the likelihood of annotating a comment as overall high-quality or uncivil across educational groups. Tables 3, 4 show the results of the logistic regression models of the different comment features and the majority votes for the educational groups.

**Table 3:** Logistic regressions for comment quality by educational group.

Predictor	Majority vote low education		Majority vote medium education		Majority vote high education	
	B	Exp (B)	B	Exp (B)	B	Exp (B)
Constant	−0.088**	**0.916**	0.130***	**1.139**	−0.091**	**0.913**
Age	−0.041	0.959	−0.015	0.986	−0.021	0.979
Comment length	1.230***	**3.420**	1.730***	**5.639**	1.768***	**5.861**
Argument	0.400***	**1.492**	0.627***	**1.873**	0.530***	**1.699**
Polite salutation	−0.565***	**0.569**	0.773***	**2.167**	−0.345**	**0.709**
Adding additional information	0.119	1.127	0.879***	**2.409**	0.851***	**2.343**
Solution proposal	0.944***	**2.569**	1.906***	**6.727**	1.467***	**4.337**
Expressing mutual respect	−0.028	0.972	0.020	1.020	0.204**	**1.227**
Personal experience/storytelling	0.245*	**1.278**	−0.536***	**0.585**	0.303*	**1.355**
Nagelkerke’s pseudo *R*^2^	0.17		0.27		0.27	
*N*	13,860		13,860		13,860	

*p < 0.05; **p < 0.01; ***p < 0.001 (in bold); calculations based on *N* = 13,860 annotations of 1,540 comments.

**Table 4:** Logistic regressions for incivility by educational group.

Predictor	Majority vote low education		Majority vote medium education		Majority vote high education	
	B	Exp (B)	B	Exp (B)	B	Exp (B)
Constant	−3.322*	**0.036**	−4.371***	**0.013**	−4.154**	**0.016**
Age	0.081	1.084	−0.163	0.849	−0.096	0.908
Comment length	−0.236**	**0.790**	−0.623***	**0.536**	−0.558***	**0.573**
Contempt	1.086***	**2.963**	1.687***	**5.403**	1.808***	**6.097**
Screaming	0.326*	**1.385**	0.443*	**1.557**	0.526**	**1.691**
Vulgarity	−0.080	0.923	0.503*	**1.653**	0.983***	**2.672**
Insults	1.126***	**3.083**	1.463***	**4.317**	1.403***	**4.067**
Accusation of lying	0.636***	**1.889**	1.211***	**3.358**	1.448***	**4.253**
Sarcasm	0.653***	**1.921**	0.850***	**2.339**	0.224*	**1.251**
Nagelkerke’s pseudo *R*^2^	0.11		0.24		0.24	
*N*	13,860		13,860		13,860	

*p < 0.05; **p < 0.01; ***p < 0.001 (in bold); Calculations based on *N* = 13,860 annotations of 1,540 comments.

#### Deliberative comment features

Overall, the presence of deliberative features in comments was generally associated with higher odds of a comment being judged as high-quality. However, when comparing the odds ratio values, it becomes clear that the three groups evaluated the presence of some deliberative features differently. For example, the occurrence of additional knowledge increased the odds of rating a comment as high-quality in the groups of medium and highly educated crowd annotators, but not in the group of lower educated annotators. Including solution proposals increased the odds of rating a comment as high-quality in all groups, but the odds were higher for the medium and highly educated groups than in the lower educated group. This was also true for lengthy comments. For some comment features, even the direction of the associations differed: Sharing personal experiences increased the likelihood of a high-quality majority vote in both the low and high education groups but decreased it in the medium education group. In contrast, the presence of a polite salutation was positively associated with the majority vote on comment quality in the medium education group but negatively associated with the respective majority votes in the low and high education groups. Overall, comparing the pseudo-*R*^2^ values of the three models, it is notable that the deliberative comment features included in the models resulted in a better model fit for explaining the quality annotations of the medium and highly educated groups compared to the lower educated group.

#### Uncivil comment features

For incivility, results show that the occurrence of uncivil features resulted in higher odds for a comment to receive an incivility majority vote across all groups, with contempt and insults having the strongest positive associations across all models. However, a comparison of the odds ratios of the three models revealed that again the occurrence of some uncivil comment features had stronger associations with the incivility annotation of medium- and highly educated groups than with those of annotators with lower levels of formal education. For instance, comments that included contempt were five and even six times more likely to be labeled as uncivil by the groups with medium or high formal education, whereas it only tripled the chances for receiving an incivility label by the annotator group with low formal education. A similar pattern was observed for accusations of lying. The occurrence of vulgarity did not impact the incivility majority vote among annotators with low formal education at all, in contrast to the other educational groups. The only exceptions from this pattern were comment length, where longer comments were less effective at reducing the odds of being labeled as uncivil by the group with low formal education compared to the other groups, and sarcasm, which was judged most rigidly by medium-educated annotators. As with the high-quality comment models, the pseudo-*R*^2^ values suggested a weaker model fit for the group of crowd workers with low formal education.

## Discussion

### Educational bias in comment quality and incivility classification?

This study investigated whether unequal representation of crowd annotators’ educational levels could be a potential bias risk for the classification of high-quality and uncivil comments in online discussions. Our findings revealed that classifiers performed differently when trained on data labeled by annotators with varying levels of formal education. For high-quality comment classification, the performances of classifiers trained on data from specific educational groups were highest when tested on data from the same group. At the same time, when applied to test sets derived from other educational groups, the classifiers’ performance decreased significantly. We observed indications for a similar pattern when comparing performance results for incivility classification. However, the robustness of the results of the incivility classifications were compromised due to the heavy imbalance in the dataset. Nonetheless, our findings reinforce the initial observations of educational bias in incivility classification reported by Al Kuwatly et al. (2020) and further, provide first empirical evidence of bias in comment quality classification, suggesting an overall group-specific annotation pattern. We further found that while deliberative comment features similarly increased the likelihood of a majority vote in favor of high comment quality across all groups, they provided a better model fit for explaining annotations by crowd annotators with medium and high levels of formal education than for those with lower educational backgrounds. Again, a similar pattern could be observed for group-specific incivility majority votes. This may indicate that comment features associated with traditional operationalizations of deliberative quality and incivility are more salient in the judgment processes of crowd annotators with medium and high levels of formal education, while being less influential for those with lower educational backgrounds.

One possible explanation of these findings is a group-specific interpretation of communicative value and perceived norm violations by formal education, which is in line with findings by Kenski et al. (2020) on varying perceptions of the severity of certain features of uncivil language. Annotators with lower levels of formal education may hold more multifaceted conceptions of comment quality and incivility that go beyond the features traditionally used to operationalize these theoretical constructs. We also found that the occurrence of some deliberative comment features (storytelling and polite salutation) in comments even showed opposing statistical associations with the majority votes of the educational groups. While this could again be a sign of an in-group-specific understanding of certain communication features, another possible explanation could be that such features can be evaluated more ambivalent in general, for example, polite salutations in online comments could be understood as sarcastic. Overall, the findings of this study suggest a risk of annotator bias caused by differing formal education in both comment quality and incivility classification in online discussions. These findings are in line with theoretical work on deliberative theory suggesting that the assessment of deliberative quality and incivility is linked to people’s educational backgrounds (Sanders, 1997; Young, 2000), arguing in favor of acknowledging different communication modes from several language styles (Barkley Brown, 1994; Edyvane, 2020; Polletta and Lee, 2006).

### Limitations

The results of this study should be critically reflected considering the following limitations: We employed a quasi-experimental train-test design to compare the performances of classifiers trained on annotations by different educational groups of annotators. While this approach is advantageous for achieving high external validity, it carries the risk of confounding factors within the compared groups: For instance, there was a small, but significant age difference for the group of medium educated crowd annotators. While this was accounted for in the logistic regression models, it could not be controlled for in the classifier comparison. Additionally, unobserved factors such as the average time spent on annotations, as well as annotators’ attention/distraction levels, may have influenced the results. Moreover, recruiting a sufficient number of participants from the lower-educated group proved challenging. For future research, we strongly recommend designing annotation tasks in ways that are equally accessible and appealing across all subgroups. Finally, the logistic regression results are based on a convenience sample and are therefore not representative of our dataset or the general population. We encourage future research to replicate both analyses using additional datasets.

It is further notable that we achieved very high average performance values, which can be partly attributed to the use of 20-fold cross-validation. This setup allowed each model to train on 95% of the data, increasing the likelihood of overfitting and inflating performance. As each fold’s validation set represents only 5% of the data, it may not fully capture the variability of the underlying distribution, further contributing to optimistic estimates. Nonetheless, the differences between annotator groups remain interpretable to a meaningful degree. Since each model is trained and evaluated within a single annotator group using a fixed random seed for fold assignment, all groups are assessed under identical and reproducible conditions. Therefore, while absolute scores may be inflated, relative performance differences likely reflect underlying variation in annotation consistency and model learnability across groups. However, for incivility, class imbalance strongly influenced the corresponding classification. In many subgroups, the positive class (*Uncivil*) was too rare to learn a reliable association pattern, resulting in degenerate predictions where the classifier predicted only the majority class (*Not Uncivil)*. Consequently, F1-scores as well as recall and precision often dropped to 0.0, as the model failed to produce any true positives for the minority class (*Uncivil*), underscoring the challenge of detecting low-prevalence phenomena like incivility (Stoll et al., 2020). Future research should address this limitation by employing stratified sampling to ensure more balanced and representative annotation subsets or by annotating substantially larger datasets, though this would entail increased resource demands. Furthermore, for each comment with high disagreement, we collected three annotations per educational group. Prior research, however, suggests that increasing the number of labels for each group and comment would be more effective in representing a broader range of relevant perspectives and stabilizing agreement levels among annotators (Chulvi et al., 2023).

Ultimately, our operationalization of formal education is limited to the highest (formal) degree in general and academic education. This approach neglects other forms of higher education such as vocational training outside academic institutions which are accounted for in the ISCED 2011 scheme. Although our focus on the highest formal degree was appropriate for this study, as informed by deliberation literature, it is important to note that holding a formal degree is only one of many ways to measure individual education success.

### Practical implications and further work

Not accounting for systematic differences in classifier performance for comment quality and incivility poses a significant risk of discrimination for AI-based moderation. ML models used to automatically identify high-quality and uncivil comments in online discussions may systematically overlook comments that deviate from the majority perspectives in the training data. This underlines the need to develop strategies on how to account for such differences in data labelling practices and model development. Based on the overall higher performances of within-group classification, our work informs the proposal to balance proportions of crowd annotators from different levels of formal education to adequately reflect different concepts of high-quality and uncivil comments. Also, using common indicators of the theoretical concepts of deliberative quality and incivility to inform training of ML models might be less fitting to reflect the perceptions of lower educated parts of the population. That raises the question on how lay-people’s understanding of comment quality differs from common scientific operationalization of deliberative quality and incivility and how we should cope with differing fits of these concepts for differing perceptions in parts of the population. Finally, the growing use of closed LLMs like OpenAI’s GPT for comment classification and moderation exacerbates challenges in identifying and addressing potential biases in moderation outputs. Recent studies show that using LLMs with zero- or few-shot learning can achieve strong performance in classifying uncivil and deliberative comments (Stoll et al., 2025; Stolwijk et al., 2025), making them increasingly attractive for moderation tasks. Beyond classification, their generative capabilities enable the drafting of responses, thereby supporting or even fully automating several steps of the moderation process (Kolla et al., 2024; Korre et al., 2025). However, this also extends the risk of educational biases to both comment selection (as inspected in this work) and response generation. At the same time, the lack of access to training data in closed models complicates efforts to systematically detect and mitigate such biases. This underscores the need for experimental setups to uncover annotator bias and develop robust mitigation strategies.

Given the limited empirical research on annotator bias in comment classification, our findings highlight the need for further investigation into comment quality and incivility as potential subjects of bias, and formal education as a possible source of such bias. Firstly, we encourage replicating these findings on different datasets, to enable further exploration of systematic disparities in training data across educational groups. For this purpose, it could be useful to add transparent information on the educational level of the annotators to dataset descriptions of crowd-annotated and openly available datasets (Bender and Friedman, 2018). Secondly, we propose investigating the underlying causes of these differences by examining systematic variations in how laypeople with different levels of formal education perceive comment quality and incivility. Our findings indicate that the importance of certain communication features differ depending on annotators’ educational backgrounds. While the traditional set of deliberative and uncivil features aligns well with respective perceptions among medium and highly educated crowd annotators, there are also signs of group-specific influences that seem to extend beyond the factors we tested, particularly among the lower educated group. To validate the assumption of differing quality and incivility perceptions of educational groups, future research would require expanding their focus beyond crowd annotators to the general population.

## Conclusion

ML classifiers are increasingly used to automatically detect high-quality and uncivil online comments for content moderation purposes. At the same time, classifiers dealing with such subjective concepts carry the risk of being impaired by annotator bias, caused by an insufficient and one-sided representation of differing perceptions across social groups. This study investigated possible annotator bias in comment classification among crowd annotators with low, medium, or high levels of formal education. Results show that classifiers for high-quality comments performed differently when trained on data labeled by annotators with different educational levels. While we see first indications for a similar pattern for incivility classification, our findings are limited by a heavy imbalance of incivility labels within our dataset. Further, the presence of fine-grained comment features linked to the theoretical constructs of deliberative quality (such as solution proposals and additional knowledge) as well as incivility (such as vulgarity, contempt, and the accusation of lying) were better suited to explain the annotations of the medium and high educational group, while being less relevant for annotators with lower formal education.

This study demonstrated that integrating conceptual and theoretical perspectives on bias in computational approaches underscores the valuable contribution of social science to NLP and ML research, particularly in uncovering bias risks in classification models arising from differing perceptions across social groups. For future research, we hope that our work contributes to a broader recognition of how biases originating in sociodemographic differences — including but not limited to educational background — can influence computational models and AI systems, and encourages future research to examine these dynamics across a wide range of classification tasks. Ultimately, we hope that the findings of this study contribute to a more nuanced and socially inclusive understanding of the value of user contributions to online discourse, helping to inform fairer data collection and annotation practices for training ML models.

## Data Availability

The datasets presented in this article are not readily available because, even when pseudonymized, they contain personal data of crowd annotators that falls under the protection of the European General Data Protection Regulation (GDPR). The full code for classifier comparison is available at: https://github.com/ankekat1000/annotator-bias-comment-classification. Requests to access the datasets should be directed to lena.wilms@hhu.de.

## Footnotes

1 Instructions are translated from German. For the original instructions in full length, please refer to the Appendix.

2 Two test annotations provided by the crowdsourcing platform were excluded from the analysis.

3 Model “bert-base-german-dbmdz-uncased.” Available open access at: https://huggingface.co/dbmdz/bert-base-german-uncased (Accessed March, 27 2026).

4 Available online at: https://github.com/ankekat1000/annotator-bias-comment-classification (anonymized for blind review).

5 Table A2 in the appendix includes a detailed description of the categories, their frequencies within the dataset, and the intercoder reliability.

6 Variables were recoded with reference to the arithmetic mean of the category within this subset: For comment length, 0 indicates a comment length below or exactly the average comment length of 65 words, 1 means a comment contains more than 65 words. For age, 0 indicates that the crowd annotator was below or exactly the average age of 40  years, crowd annotators older than 40  years were recoded as 1.

7 The reported differences are significant according to the Kolmogorov-Smirnov test of equality of distributions on two samples. Details on the calculations and results can be found in the source-code provided: https://github.com/ankekat1000/annotator-bias-comment-classification.

8 The reported differences are significant according to the Kolmogorov-Smirnov test of equality of distributions on two samples. Details on the calculations and results can be found in the source-code provided: https://github.com/ankekat1000/annotator-bias-comment-classification.
