# Supplementary material for: Investigating annotator bias in comment quality and incivility classification by formal education
Source: Front Artif Intell. 2026 Jun 1;9:1772844. doi: 10.3389/frai.2026.1772844 (PMC13267338; doi:10.3389/frai.2026.1772844)
Supplement: Supplementary file 1 [file Data_Sheet_1.pdf]

This appendix contains supplementary material to the research article “Investigating annotator bias in comment quality and incivility classification by formal education” by Lena Wilms, Anke Stoll and Marc Ziegele, published in Frontiers in Artificial Intelligence.

Appendix 1

Table A1

Crowd Annotator Information Overview

	Low education	Medium education	High education	Total
$N_{\text{Participants}}$	197	228	256	681
average no. of annotations per individual annotator	*M* = 207, *SD* = 182	*M* = 178, *SD* = 141	*M* = 160, *SD* = 104	*M* = 180, *SD = 143*
$N_{\text{Annotations}}$	41,030	41,030	41,031	123,091
age	*M* = 41, *SD* = 13	*M* = 36, *SD*= 12	*M* = 42, *SD* = 12	*M* = 40, *SD* = 12
percentage male gender (compared to female or diverse)	59%	57%	63%	60%
most indicated highest degree in general/academic education	general or intermediate education up to grade 10	completion of intermediate schools up to grade 13	academic degree	academic degree
median frequency of reading comments in online discussions*	*Md* = 5 (reads comments daily)	*Md* = 5 (reads comments daily)	*Md* = 5 (reads comments daily)	*Md* = 5 (reads comments daily)
median frequency of writing comments in online discussions*	*Md* = 3 (writes comments once or more per month)	*Md* = 2 (writes comments less than once per month)	*Md* = 3 (writes comments once or more per month)	*Md* = 3 (writes comments once or more per month)

Note: *Ordinal scale between -6 (1 = never reads/writes comments; 6 = reads/writes comments daily).

Table A2

Coding Scheme and Dataset Metrics of the Annotated Subset

Category and description	*N* (%)	Krippendorff's alpha
Argument<br>Statements to substantiate or refute opinions	476 (30.9 %)	0.73
Adding additional information<br>Additional information is cited as references for opinion	129 (8.4 %)	0.72
Solution proposal<br>Constructive solution proposals are democratic, realistic and rational in the broadest sense	400 (26.0 %)	0.75
Polite salutation<br>Use of polite language indicated by e.g., polite salutation	45 (2.9 %)	0.80
Expressing mutual respect<br>Giving credit to accomplishments or praising personality traits of another person	156 (10.1 %)	0.71
Personal experience/Storytelling<br>Sharing personal stories or experiences	57 (3.7 %)	0.85
Contempt<br>derogatory, disrespectful, and/or disparaging remarks about persons, groups, institutions, or ideas	303 (19.7 %)	0.81
Screaming<br>Implying volume by writing in all-caps	87 (5.6 %)	0.76
Vulgarity<br>Use of obscene, foul, or boorish language	25 (1.6 %)	0.71
Insults<br>Swear words and derogatory statements	34 (2.2%)	0.56
Accusation of lying<br>Insinuation that ideas, plans, actions or policies are dishonest, subterfuge and misleading	61 (4.0 %)	0.72
Sarcasm<br>Ruthless, biting mockery	216 (14.0 %)	0.68

Notes: Manually coded subsample of *N*= 1,540 comments from a citizen participation project in Germany. (Ordinal) Krippendorff's alpha was calculated between three annotators on a subset of 159 comments. Original variables were measured on a 4-point scale (Heinbach & Wilms, 2022).

Table A3

Performance Results for Comment Quality Classification

Train set \ Test set		Low Education	Medium Education	High Education	Mixed
Low Education	Macro-avg. F1	**0.97**	0.74	0.73	0.78
	Accuracy	**0.97**	0.75	0.74	0.8
	Recall	**0.98**	0.7	0.72	0.77
	Precision	**0.98**	0.77	0.74	0.8
Medium Education	Macro-avg. F1	0.75	**0.96**	0.78	0.8
	Accuracy	0.76	**0.96**	0.78	0.8
	Recall	0.8	**0.98**	0.8	0.84
	Precision	0.71	**0.95**	0.75	0.78
High Education	Macro-avg. F1	0.71	0.78	**0.92**	0.73
	Accuracy	0.76	0.79	**0.93**	0.79
	Recall	0.74	0.76	**0.92**	0.73
	Precision	0.72	0.81	**0.96**	0.76
Mixed	Macro-avg. F1	0.78	0.82	0.81	**1.0**
	Accuracy	0.79	0.82	0.82	**1.0**
	Recall	0.81	0.80	0.82	**1.0**
	Precision	0.76	0.84	0.81	**1.0**

Notes. Performance scores are averaged values of 20 runs (20-fold cross validation). N_{train} = 12,993 and N_{test} = 684 comments in each run. Performances over 0.9 are highlighted in bold. Recall and precision are reported for the positive class (*High Quality*).

Table A4

Performance Results for Incivility Classification

Train set \ Test set		Low Education	Medium Education	High Education	Mixed
Low Education	Macro-avg. F1	**0.95**	0.5	0.49	0.49
	Accuracy	**0.99**	0.88	0.87	0.9
	Recall	**0.93**	0.53	0.51	0.49
	Precision	**0.96**	0.47	0.47	0.51
Medium Education	Macro-avg. F1	0.38	0.6	0.33	0.0
	Accuracy	**0.9**	**0.94**	0.89	0.89
	Recall	0.32	0.54	0.31	0.0
	Precision	0.67	0.7	0.37	0.0
High Education	Macro-avg. F1	0.0	0.0	0.0	0.0
	Accuracy	0.87	0.89	0.88	0.89
	Recall	0.0	0.0	0.0	0.0
	Precision	0.0	0.0	0.0	0.0
Mixed	Macro-avg. F1	0.0	0.0	0.0	0.0
	Accuracy	0.87	0.89	0.88	0.89
	Recall	0.0	0.0	0.0	0.0
	Precision	0.0	0.0	0.0	0.0

Notes. Performance scores are averaged values of 20 runs (20-fold cross-validation). N_{train} = 12,993 and N_{test} = 684 comments in each run. Performances over 0.9 are highlighted in bold. Recall and precision are reported for the positive class (*Uncivil*).

Appendix 2

Study material documentation:

This appendix documents the study materials used in the crowdworker experiment. It provides a full textual reproduction of the original German-language instructions shown to participants, including (1) the task description, (2) category definitions with illustrative annotation examples, and (3) the socio-demographic questionnaire used for participant screening. To ensure accessibility for non-German-speaking readers, concise English summaries are provided for each section.

(1) Kategorienbeschreibung [Category Description]:

Unangemessen

Der Kommentar ist ganz oder teilweise unangemessen. Damit meinen wir Kommentare, die unhöflich, beleidigend oder hasserfüllt sind oder in anderer Weise dazu beitragen, dass sich User respektlos behandelt fühlen.

Bereichernd

Der Kommentar ist ganz oder teilweise bereichernd. Damit meinen wir Kommentare, die Argumente, Vorschläge oder neue Perspektiven in die Diskussion einbringen oder in anderer Weise dazu beitragen, dass User sie anregend oder wertschätzend finden.

Tatsachenbehauptung

Der Kommentar enthält eine Tatsachenbehauptung. Damit meinen wir Aussagen mit Wahrheitsanspruch, die geprüft, bewiesen oder widerlegt werden können. Tatsachenbehauptungen können außerdem Quellen, Belege, Links oder Zitate enthalten.

Achtung: Kommentare können gleichzeitig mehrere Kategorien enthalten.

Beispiele:

Hier finden Sie Beispielkommentare mit Musterlösung.

Beispielkommentar 1:

Diese Flüchtlinge sind Dreckspack! Die können doch nichts, außer sich hier durchschnorren. Verpisst euch!!!

Ist der Kommentar ganz oder teilweise **unangemessen**?

Ja ☒ Nein ☐

Ist der Kommentar ganz oder teilweise **bereichernd**?

Ja ☐ Nein ☒

Enthält der Kommentar eine **Tatsachenbehauptung** und/oder Belege und Quellen?

Ja ☐ Nein ☒

Beispielkommentar 2:

Danke, dass so viele Lehrer, die an der Front arbeiten eingeladen wurden um mal zu berichten, wie es wirklich aussieht und mit welchen begrenzten Mitteln gearbeitet werden muss.

Ist der Kommentar ganz oder teilweise **unangemessen**?

Ja ☐ Nein ☒

Ist der Kommentar ganz oder teilweise **bereichernd**?

Ja ☒ Nein ☐

Enthält der Kommentar eine **Tatsachenbehauptung** und/oder Belege und Quellen?

Ja ☐ Nein ☒

Beispielkommentar 3:

Kinder werden nicht nur seltener krank, sie infizieren sich wohl auch seltener mit dem Coronavirus als ihre Eltern - das ist laut Ministerpräsident Winfried Kretschmann (Grüne) das Zwischenergebnis einer Untersuchung der Unikliniken Heidelberg, Freiburg und Tübingen.

Ist der Kommentar ganz oder teilweise **unangemessen**?

Ja ☐ Nein ☒

Ist der Kommentar ganz oder teilweise **bereichernd**?

Ja ☒ Nein ☐

Enthält der Kommentar eine **Tatsachenbehauptung** und/oder Belege und Quellen?

Ja ☒ Nein ☐

Beispielkommentar 4:

Jippie, ich liebe Sommerferien! <3

Ist der Kommentar ganz oder teilweise **unangemessen?**

Ja ☐ Nein ☒

Ist der Kommentar ganz oder teilweise **bereichernd?**

Ja ☐ Nein ☒

Enthält der Kommentar eine **Tatsachenbehauptung** und/oder Belege und Quellen?

Ja ☐ Nein ☒

Beispielkommentar 5:

So einen Quatsch habe ich lange nicht mehr gehört! Wir brauchen einfach ein Bewusstsein dafür, dass Fleisch nur einmal die Woche auf den Tisch kommt. Bei uns zu Hause war das immer so und wir hatten nicht viel. So kann das Geld auch bei Geringverdienern für gutes Fleisch ausreichen.

Ist der Kommentar ganz oder teilweise **unangemessen**?

Ja ☒ Nein ☐

Ist der Kommentar ganz oder teilweise **bereichernd**?

Ja ☒ Nein ☐

Enthält der Kommentar eine **Tatsachenbehauptung** und/oder Belege und Quellen?

Ja ☐ Nein ☒

Beispielkommentar 6:

So was kann auch nur jemand behaupten, der nicht zum Denken fähig ist. Wacht endlich auf! Experten schlagen mittlerweile Alarm, denn Kinder werden mit den Folgen des Home Schooling alleine gelassen! https://www.n-tv.de/panorama/Schuetzt-Deutschland-Altere-mehrals-Kinder-article21806832.html

Ist der Kommentar ganz oder teilweise **unangemessen?**

Ja ☒ Nein ☐

Ist der Kommentar ganz oder teilweise **bereichernd?**

Ja ☐ Nein ☒

Enthält der Kommentar eine **Tatsachenbehauptung** und/oder Belege und Quellen?

Ja ☒ Nein ☐

Beispielkommentar 7:

Bitte insgesamt weniger Unterhaltung (insbesondere Quizshows und Krimis), dafür mehr Information. Viele Informationssendungen sind in den vergangenen Jahren gekürzt oder sogar eingestellt worden. Dabei bräuchten wir gerade in der heutigen Zeit mehr wissenschaftlich fundierte Information als je zuvor.

Ist der Kommentar ganz oder teilweise **unangemessen**?

Ja ☐ Nein ☒

Ist der Kommentar ganz oder teilweise **bereichernd**?

Ja ☒ Nein ☐

Enthält der Kommentar eine **Tatsachenbehauptung** und/oder Belege und Quellen?

Ja ☒ Nein ☐

English summary: This section explains the classification task given to crowdworkers. Participants were asked to evaluate online comments based on three non-exclusive categories: Inappropriate (e.g., offensive, insulting, or disrespectful content), enriching (e.g., constructive contributions, arguments, or new perspectives), and factual claim (e.g., verifiable statements that may include evidence or sources). Crowdworkers were informed that a single comment could belong to multiple categories simultaneously. It also provides several example comments with correct annotations to illustrate how the categories should be applied.

Kategorienabfrage [Annotation Task Interface]:

Bitte bewerten Sie den folgenden Text:

< Platzhalter Beispielkommentar >

Hier sehen Sie eine Auswahl von Kommentaren aus Online-Diskussionen zu verschiedenen politischen Themen. **Bitte lesen Sie die folgenden Kommentare gründlich und kreuzen Sie an.**

Ist der Kommentar ganz oder teilweise **unangemessen**?
Der Kommentar ist ganz oder teilweise unangemessen. Damit meinen wir Kommentare, die unhöflich, beleidigend oder hasserfüllt sind oder in anderer Weise dazu beitragen, dass sich User respektlos behandelt fühlen.

Ja ☐ Nein ☐

Ist der Kommentar ganz oder teilweise **bereichernd**?
Der Kommentar ist ganz oder teilweise bereichernd. Damit meinen wir Kommentare, die Argumente, Vorschläge oder neue Perspektiven in die Diskussion einbringen oder in anderer Weise dazu beitragen, dass User sie anregend oder wertschätzend finden.

Ja ☐ Nein ☐

Enthält der Kommentar eine **Tatsachenbehauptung** und/oder Belege und Quellen?
Der Kommentar enthält eine Tatsachenbehauptung. Damit meinen wir Aussagen mit Wahrheitsanspruch, die geprüft, bewiesen oder widerlegt werden können. Tatsachenbehauptungen können außerdem Quellen, Belege, Links oder Zitate enthalten.

Ja ☐ Nein ☐

Hinweis: Kommentare können auch keine sowie mehrere Kategorien gleichzeitig enthalten.

English summary: This section shows the actual task format presented to participants. Workers were asked to read individual comments from online political discussions and indicate (yes/no) whether each comment is inappropriate, is enriching, and/or contains a factual claim or supporting evidence. It reiterates the definitions and emphasizes that multiple or no categories can apply.

<u>Abfrage Sozio-Demografie und Randomisierungscheck [Participant Screening]:</u>

Frage 1: Alter

Bitte geben Sie Ihren Geburtsmonat und Ihr Geburtsjahr an.

Monat: xx* Jahr: xxxx**

Erlaubter Wertebereich: *0-12; **1900-2014

Frage 2: Geschlecht

Sind Sie…

männlich ☐ weiblich ☐ divers ☐

Frage 3: Höchster allgemeiner Schulabschluss (Schneider, 2016)

3a. Haben Sie einen allgemeinen Schulabschluss?

Ja ☐ nein/noch nicht ☐

3b. Welchen **höchsten** Schulabschluss haben Sie?

Hinweis: Ordnen Sie bitte im Ausland erworbene Abschlüsse einem gleichwertigen deutschen Abschluss zu.

Haupt-/Volksschulabschluss: ☐
Polytechnische Volkshochschule der DDR mit Abschluss der 9. Klasse: ☐
Polytechnische Volkshochschule der DDR mit Abschluss der 10. Klasse: ☐
Realschule/Mittlere Reife: ☐
Fachhochschulreife: ☐
Abitur (Allgemeine oder fachgebundene Hochschulreife): ☐
Nicht zutreffend: ☐

Frage 4: Höchster Ausbildungsabschluss (Schneider, 2016)

4a. Haben Sie einen beruflichen Ausbildungsabschluss oder einen Hochschul-/Fachhochschulabschluss?

Ja ☐ nein/noch nicht ☐

4b. Welchen höchsten Abschluss haben Sie?

Beruflicher Ausbildungsabschluss:

Anlernausbildung/berufliches Praktikum: ☐
Berufsvorbereitungsjahr: ☐
Lehre/ Berufsausbildung im dualen System: ☐
Berufsqualifizierender Abschluss in einer Berufsfachschule/ Kollegschule: ☐
Vorbereitungsdienst für den mittleren Dienst in der öffentlichen Verwaltung: ☐
Ausbildungsstätten für Gesundheits- und Sozialberufe:
… einjährig: ☐
… zweijährig: ☐
… dreijährig: ☐
Ausbildungsstätten für Erzieherinnen: ☐
Meister/-in, Techniker/-in oder gleichwertiger Fachschulabschluss: ☐
Fachschule der DDR: ☐
Fachakademie (nur in Bayern): ☐

Hochschulen/Fachhochschulen:

Diplom, Bachelor, Master, Magister, Staatsprüfung, Lehramtsprüfung:

Berufsakademie ☐
Verwaltungsfachhochschule: ☐
Fachhochschule (FH, auch Ingenieur-schule, Hochschule für angewandte Wissenschaften) ☐
Duale Hochschule Baden-Württemberg: ☐
Universität (wissenschaftliche Hochschule, auch: Kunsthochschule, Pädagogische Hochschule, Theologische Hochschule) ☐

Nicht zutreffend: ☐

4c. Wie ist die Bezeichnung ihres höchsten Abschlusses?

Bachelor: ☐
Master: ☐
Diplom/Lehramtsprüfung/Staatsprüfung/Magister/künstlerischer Abschluss oder vergleichbare Abschlüsse: ☐
Nicht zutreffend: ☐

Frage 5: Politisches Interesse (Jackob et al., 2023)

Wie interessiert sind Sie im Allgemeinen an Politik?

Sehr interessiert	☐
Eher interessiert	☐
Teilweise interessiert	☐
Eher nicht interessiert	☐
Überhaupt nicht interessiert	☐

Frage 6: Nutzeraktivität (Jackob et al., 2023)

Im Internet kann man Nachrichten in Online-Foren oder sozialen Netzwerken kommentieren. Bitte sagen Sie mir, wie häufig Sie die folgenden Aktivitäten ausführen:

6a. Kommentare anderer Nutzer lesen:

Mehrmals am Tag	☐
Täglich	☐
Ein- oder mehrmals in der Woche	☐
Ein- oder mehrmals im Monat	☐
Seltener als einmal im Monat	☐
Nie	☐

6b. Eigene Kommentare schreiben:

Mehrmals am Tag	☐
Täglich	☐
Ein- oder mehrmals in der Woche	☐
Ein- oder mehrmals im Monat	☐
Seltener als einmal im Monat	☐
Nie	☐

Frageformulierungen sind angelehnt an:

Jackob, N., Schultz, T., Jakobs, I., Quiring, O., Schemer, C., Ziegele, M., & Viehmann, C. (2023). *Medienvertrauen in Deutschland*. Bundeszentrale für politische Bildung.

Schneider, S. L. (2016). Die Konzeptualisierung, Erhebung und Kodierung von Bildung in nationalen und internationalen Umfragen. *GESIS Survey Guidelines*. https://doi.org/10.15465/GESIS-SG_020-1

English summary: This section describes the background survey used to collect participant characteristics and check randomization. It includes age, gender, educational background (general school and professional/higher education), political interest, and participation behavior in online discussions (frequency of reading and writing comments).
